# Community‐Engaged Research Methods and Preliminary Findings: Insights from ADNI4 Engagement Core's Community Research Liaisons and Navigators

**DOI:** 10.1002/alz70860_099540

**Published:** 2025-12-23

**Authors:** Jacqueline Pontes Monteiro, Hannatu Amaza, Isabella Hoang, Alyssa Arentoft, Vanessa A. Guzman, Lauren Smitherman, Zipporah Melton, Chester DeWitt, Natalie Perez, Mariana Perez, Joseph DiBenedetto, Fabiola Magaña, Lisa Renier Thomas, Shaniya Parkins, Julianne Yoon, Caterina Bocardo, Matt Glittenberg, Stephen Cubbellotti, Adam Diaz, Laura Wang, Michael W Weiner, Rachel L. Nosheny, Monica G Rivera Mindt, Ozioma C. Okonkwo, Alzheimer's Disease Neuroimaging Initiative (ADNI)

**Affiliations:** ^1^ Wisconsin Alzheimer's Disease Research Center, School of Medicine and Public Health, Madison, WI, USA; ^2^ Wisconsin Alzheimer's Disease Research Center, School of Medicine and Public Health, University of Wisconsin‐Madison, Madison, WI, USA; ^3^ California State University Northridge, Northridge, CA, USA; ^4^ Icahn School of Medicine at Mount Sinai, New York, NY, USA; ^5^ Georgetown University Medical Center, Washington, DC, USA; ^6^ Goodman Hall Neuroscience Center, Indianapolis, IN, USA; ^7^ Butler Hospital Memory and Aging Program, Providence, RI, USA; ^8^ University of California, Los Angeles, CA, USA; ^9^ University of California, Davis, CA, USA; ^10^ Wisconsin Alzheimer's Disease Research Center, School of Medicine and Public Health, University of Wisconsin‐Madison, Madison, WI, USA; ^11^ University of California, San Francisco, San Francisco, CA, USA; ^12^ Duke University Medical Center, Durham, NC, USA; ^13^ Northern California Institute for Research & Education (NCIRE), San Francisco, CA, USA; ^14^ University of California San Francisco (UCSF), San Francisco, CA, USA; ^15^ Fordham University, New York, NY, USA

## Abstract

**Background:**

A critical goal of ADNI4's Engagement Core is to promote engagement to ensure the generalizability of ADNI. To this end, ADNI4 Engagement Core employs a multi‐pronged community‐engaged research (CER) model. We highlight components of this model and provide enrollment updates to assess the preliminary efficacy of this approach.

**Method:**

Participants include persons from low education, rural, and other backgrounds. To promote engagement 1) our CER model prioritizes trust‐building, co‐learning, power‐sharing, fostering sustainable community partnerships with community‐based organizations (CBOs) and participants, across ADNI4's digital, blood biomarker, and in‐clinic phases; 2) we partner with “Hub Sites” which receive support for participant engagement, including a funded, full‐time Community Research Liaison (CRL). CRLs are our “boots on the ground.” They work with CBOs (e.g., social services, faith groups) and community members to build partnerships, participate in events, share accurate information, assess community needs, and address beliefs to improve messaging; 3) we deploy Community Research Navigators (CRNs) who work remotely (e.g., phone, email, chat), to apply CER principles to support participants and study partners, address technology/logistical issues, answer inquiries, and nurturing relationships.

**Result:**

As of January 2025, there are 10 Hub Sites, 6 CRLs, and 6 CRNs. CRLs engaged 85 CBOs and participated in 101 community events (e.g., educational lectures, focus groups), reaching 7,994 people. CRNs have created 3,124 tickets (communications) responding to community members questions/concerns all over the U.S. Most communications have been via email (56%) or phone (42%). Leading topics include blood biomarkers, enrollment, and study questions/payments. Of the *new in‐clinic* enrollees we reached our goal of greater generalizability. Our 10 Hub Sites (∼20% of ADNI sites), accounted for ∼50% of the improved generalizability.

**Conclusion:**

ADNI4 has scaled a comprehensive CER model to the national level. These preliminary findings suggest these methods are resulting in increased engagement and generalizability, exceeding our goal. These efforts require substantial support and resources and focus on building trust and long‐term community partnerships. These strategies provide a replicable, scalable model for more generalizable data for AD/ADRD field.

